# Distinct asciminib sensitivity profiles between BCR::ABL1 p190 and p210 isoforms

**DOI:** 10.1186/s40164-026-00859-0

**Published:** 2026-11-11

**Authors:** Elias Lagonik, Luke J. Quinlan, Elyse C. Page, Daniel P. McDougal, Laura N. Eadie, John B. Bruning, David T. Yeung, Timothy P. Hughes, Deborah L. White

**Affiliations:** 1 Precision Cancer Medicine Theme, South Australian Health and Medical Research Institute, Adelaide, SA Australia; 2 Faculty of Sciences, Engineering and Technology, The University of Adelaide, Adelaide, SA Australia; 3 Faculty of Health and Medical Sciences, The University of Adelaide, Adelaide, SA Australia; 4 Centre for Targeted Protein Degradation, University of Dundee, Dundee, Scotland; 5 Institute for Photonics and Advanced Sensing, School of Biological Sciences, The University of Adelaide, Adelaide, SA Australia; 6 Haematology Department, Royal Adelaide Hospital, Adelaide, SA Australia; 7 Australasian Leukaemia Lymphoma Group, Richmond, VIC Australia; 8 Australian and New Zealand Children’s Haematology-Oncology Group, Clayton, VIC Australia

**Keywords:** CML, Ph + ALL, BCR::ABL1, p190, T315I, STAMP, Asciminib, TERN-701, MK-4208, Resistance

## Abstract

BCR::ABL1 is the oncogenic driver of chronic myeloid leukemia (CML) and Philadelphia chromosome-positive (Ph + ) acute lymphoblastic leukemia (ALL), with BCR::ABL1 p210 and p190 being the predominant isoforms. Asciminib, an inhibitor that specifically targets the ABL myristate pocket (STAMP), has demonstrated efficacy in both pre-clinical and clinical studies against p210-driven CML, including cases where resistance mediated by ATP-pocket mutations has developed during prior tyrosine kinase inhibitor (TKI) therapy. The resistant mutation profile of asciminib is currently considered largely distinct from that of ATP-competitive TKIs. In our study, we demonstrate that several ATP-pocket mutations, including the V299L, F311I, T315I, and F317C/L, confer high levels of resistance to asciminib in the p190 isoform (> 1000-fold increase in LD_50_^asciminib^ values compared to non-mutated BCR::ABL1 p190), but remain sensitive when occurring in the p210 isoform. The T315A was the only tested mutation in BCR::ABL1 p190 sensitive to asciminib. Furthermore, cells expressing BCR::ABL1 p190 mutations exhibited decreased kinase inhibition following STAMP treatment, indicative of an active kinase. Analogous results were obtained for MK-4208 (formerly known as TERN-701), a clinically active investigational STAMP compound. In the BCR::ABL1 p190 setting, this work reveals an overlapping resistance profile between STAMP compounds and TKIs, with important considerations for STAMP-based therapy. It challenges two key assumptions: that asciminib dose escalation can overcome resistant ATP-pocket mutations and that targeted therapy sensitivity profiles are equivalent between the p190 and p210 isoforms.

**Supplementary Information:**

The online version contains supplementary material available at https://doi.org/10.1186/s40164-026-00859-0.

BCR::ABL1 drives both Chronic Myeloid Leukemia (CML) and Philadelphia-positive (Ph + ) Acute Lymphoblastic Leukemia (ALL). The two major isoforms are the p190 (~ 75% of Ph + ALL) and p210 (Fig. 1A) [1]. While tyrosine kinase inhibitors (TKIs) have significantly improved patient outcomes, TKI-resistance due to BCR::ABL1 mutations remains a therapeutic challenge [1, 2]. In Ph + ALL, p190 patients have a greater variety of mutations, with second-generation TKIs effectively overcoming most mutations (Fig. 1B; Supplemental Fig. S1) [1, 3]. An exception is the gatekeeper T315I, frequently detected at relapse, which requires ponatinib, a third-generation TKI whose clinical use is limited by cardiotoxicity [1, 4]. For ATP-competitive TKIs, the sensitivity profiles of a given mutation are assumed to be equivalent between BCR::ABL1 p190 and p210 isoforms. Consequently, BCR::ABL1 isoform status has not been considered in dictating therapeutic decisions [1, 4]. Here, we challenge this assumption in the context of allosteric inhibitors.

**Fig. 1:**
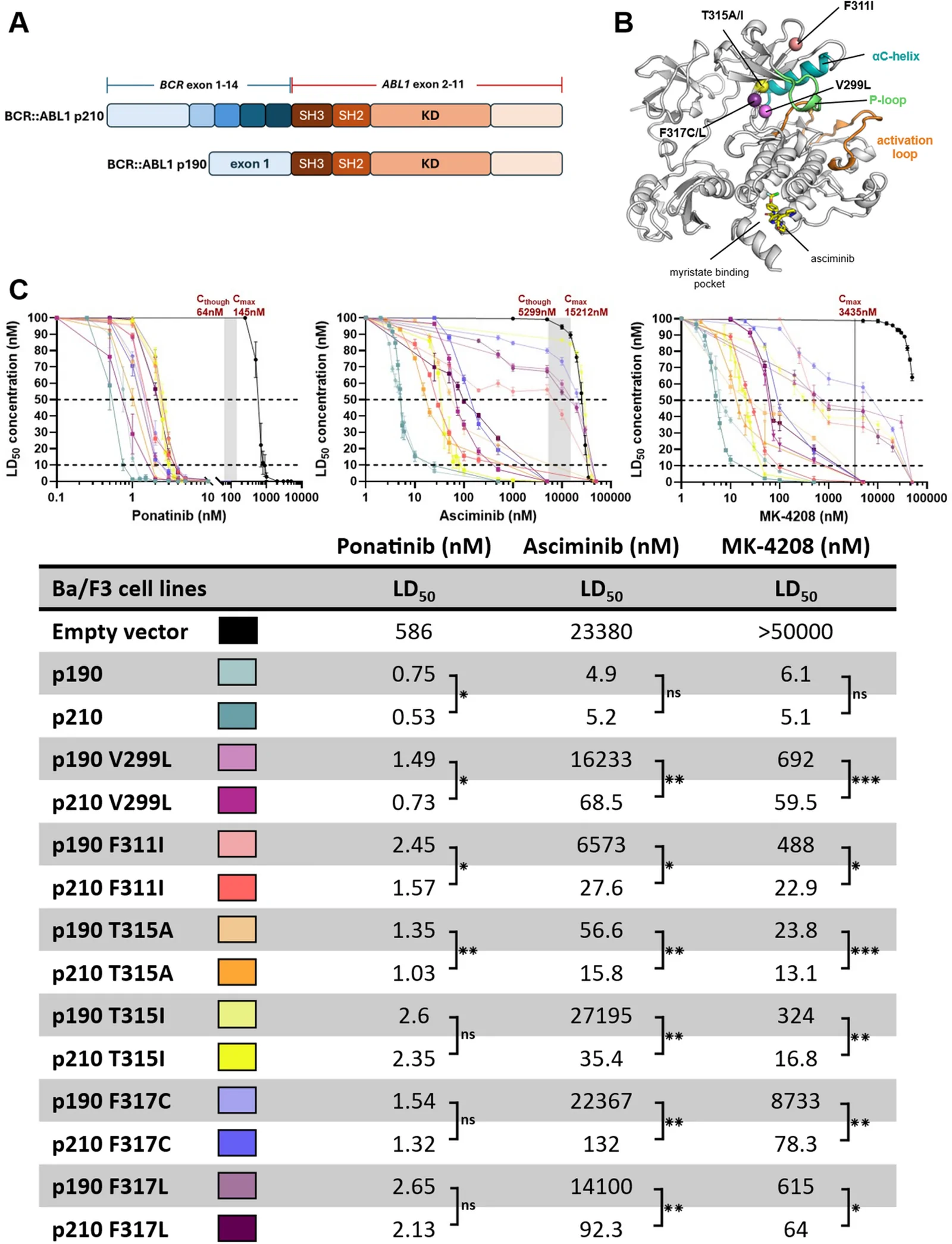
Schematic representation of BCR::ABL1 p190 and p210 isoforms and their inhibitor sensitivity (LD_50_) profiles. **A** Overview of BCR::ABL1 p190 and p210 isoforms and **B** cartoon representation of the location of the investigated ATP-pocket mutations in ABL1 (PDB: 1OPL). P-loop (green), αC-helix (blue), and activation loop (orange) elements are highlighted. **C** In vitro analysis of viability of Ba/F3 cells expressing the indicated BCR::ABL1 mutations after treatment with ponatinib, asciminib and MK-4208. All experiments were performed in biological replicates (n ≥ 3). The mean LD_50_ (nanomolar) concentration for each cell line is shown. Grey shaded areas represent the steady-state maximal plasma concentration of ponatinib (45 mg once daily): C_max_ = 145 nM & C_trough_ = 64 nM [4], asciminib (200 mg twice daily): C_max_ = 15​212 nM & C_trough_ = 5299 nM [2], and MK-4208 (320 mg once daily): C_max_ = 3435 nM (personal communication). Statistical significance was calculated by Unpaired Student’s t-test with Welch’s correction (*p < 0.05, **p < 0.01, ***p < 0.001, ns = not significant, SH2 = Src homology 2, SH3 = Src homology 3, KD = kinase domain)

Asciminib was developed to overcome ATP-pocket mutations by specifically targeting the ABL myristate pocket (STAMP) and locking the kinase into an inactive conformation [5, 6]. While asciminib is effective in most patients with BCR::ABL1 p210 ATP-pocket mutations, [2, 5, 7] evaluation in Ph + ALL remains limited. In a phase I clinical study (NCT02081378) of asciminib monotherapy (40 to 280 mg twice-daily), responses were transient and all TKI-resistant patients (n = 28) discontinued therapy, primarily due to disease progression (n = 13; 46.4%)​ [8]. The efficacy results were inconclusive because the trial was not designed to evaluate asciminib efficacy against specific isoforms o​r mutations. In another study (NCT03595917), asciminib combined with dasatinib and corticosteroids achieved complete hematological remission in de novo Ph + ALL patients (n = 22/22). However, four patients relapsed, with one acquiring only the T315I mutation [9]. 

Preclinical studies assessing the potency of asciminib and MK-4208 (clinically active STAMP compound) against BCR::ABL1 p190 mutations are critical for their clinical translation and form the basis of this study.​​ Inhibitor efficacy and kinase inhibition were evaluated in Ba/F3 cells expressing mutations in p190 or p210 isoforms (Supplemental Figs. S2, S3). Concentrations were compared to steady-state maximal/minimal drug plasma concentrations [2, 4].

In our study, no difference in asciminib efficacy between non-mutated BCR::ABL1 p190 and p210 isoforms was observed, while BCR::ABL1 p210 mutations exhibited reduced sensitivity, consistent with previous studies (Fig. 1C) [5, 7]. Interestingly, identical mutations in the p190 isoform conferred high degrees of resistance to asciminib (LD_50_^asciminib^ concentrations > 5000 nM). Among these was T315I (present in the majority of relapsed Ph + ALL) [3], which exhibited a > 5000-fold increase in LD_50_^asciminib^ concentration compared to non-mutated p190 and a > 700-fold increase compared to p210 T315I. Interestingly, the p190 T315A was the only mutation with an LD_50_^asciminib^ in the nanomolar range (Fig. 1C), highlighting that not all ATP-pocket mutations in p190 are resistant to asciminib. MK-4208 was more potent than asciminib as measured by the LD_50_ concentrations. Importantly, LD_90_ concentrations were similar between allosteric inhibitors (Supplementary Table 4) and normalization to BCR::ABL1 protein expression did not affect LD_50_ concentrations (Supplementary Table 5). ATP-competitive TKIs showed < 1-fold increase in LD_50_ values for p190 cell lines, with ponatinib remaining effective against all BCR::ABL1 mutations (Fig. 1C, Supplemental Fig. 4). Furthermore, Ba/F3 cells harboring the investigated mutations in the p190 isoform exhibited decreased kinase inhibition (higher levels of pSTAT5) following STAMP treatment compared to p210. Ponatinib treatment inhibited kinase activity regardless of mutation or isoform (Fig. 2). Importantly, kinase inhibition results confirmed our LD_50_ findings and are consistent with an active BCR::ABL1 p190 oncoprotein.

**Fig. 2:**
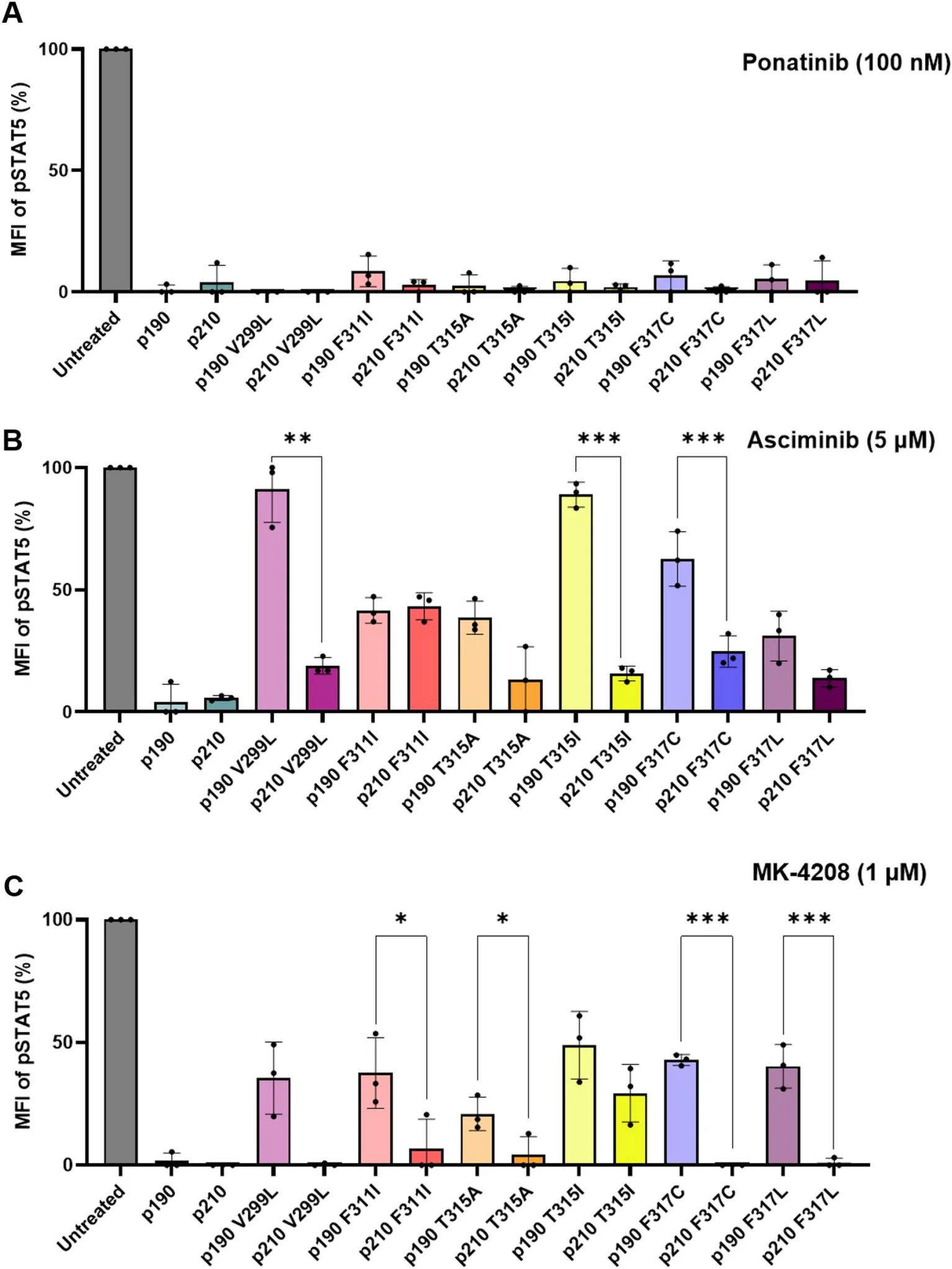
BCR::ABL1 p190 mutations exhibit lower kinase inhibition after STAMP treatment compared to identical BCR::ABL1 p210 mutations. Intracellular flow cytometric assessment of kinase inhibition in Ba/F3 cells expressing the indicated BCR::ABL1 p190 or p210 mutations. Cell lines were treated with **A** ponatinib (100 nM), **B** asciminib (5 μM), and **C** MK-4208 (1 μM). Experiments were performed in biological replicates (n = 3). Treatment-induced kinase inhibition in BCR::ABL1 p190 mutation cell lines was compared with that of the corresponding mutations in p210 for each drug. MFI pSTAT5 was normalized to the DMSO control (MFI = Mean Fluorescent Intensity). Columns represent mean ± SD. Statistical significance was calculated by Unpaired Student’s t-test with Welch’s correction (*p < 0.05, **p < 0.01, ***p < 0.001)

Our data demonstrated that STAMP inhibitors are ineffective against BCR::ABL1 p190 T315I and other ATP-pocket mutations, with recent data also suggesting that P-loop mutations Y253H and E255V confer resistance to asciminib [10]. Further validation of asciminib efficacy across a broader panel of BCR::ABL1 p190 mutations and in human cell lines or primary Ph + ALL cells is warranted.

We hypothesise that the p190 isoform's unique features, in the presence of ATP-pocket mutations, might negatively impact the STAMP-mediated BCR::ABL1 inactive conformation [6, 7, 11]. Beyond intramolecular interactions, BCR::ABL1 p190 isoform-specific pathways may drive overexpression or hyperphosphorylation of ABL1 interactor proteins. These proteins can displace the SH2-SH3 domain inhibitory mechanism, further impairing STAMP efficacy [12]. While these data have inherent limitations for clinical interpretation, the findings suggest using STAMP compounds with caution in high-risk BCR::ABL1 p190 patients with ATP-pocket mutations. The findings also directly challenge the assumption that targeted therapy sensitivity profiles are convertible between p190 and p210 isoforms.

## Supplementary Information

Additional file 1.

## Data Availability

Data are available by emailing the corresponding author, Prof. Deborah White (deborah.white@sahmri.com).

## Abbreviations

CML: Chronic myeloid leukemia
Ph +: Philadelphia positive
ALL: Acute lymphoblastic leukemia
TKI: Tyrosine kinase inhibitor
STAMP: Specifically targeting the ABL myristate pocket
LD: Lethal dose
