# Supplementary material for: Distinct asciminib sensitivity profiles between BCR::ABL1 p190 and p210 isoforms
Source: Exp Hematol Oncol. 2026 Nov 11;15:122. doi: 10.1186/s40164-026-00859-0 (PMC13753438; doi:10.1186/s40164-026-00859-0)
Supplement: Supplementary file 1 [file 40164_2026_859_MOESM1_ESM.docx]

**SUPPLEMENTAL APPENDIX**

**Distinct asciminib sensitivity profiles between BCR::ABL1 p190 and p210 isoforms**

**Running title:** p190 ATP pocket mutations are resistant to asciminib

Elias Lagonik^1,2^, Luke J. Quinlan^1,3^, Elyse. C. Page^1,3^, Daniel P. McDougal^2,4^, Laura N. Eadie^1,3^, John B. Bruning^2,4^, David T. Yeung^1,3,5,6^, Timothy P. Hughes^1,3,5,6^, Deborah L. White^1,2,3,6,7^.

^1^Precision Cancer Medicine Theme, South Australian Health and Medical Research Institute, Adelaide, SA, Australia.

^2^Faculty of Sciences, Engineering and Technology, The University of Adelaide, Adelaide, SA, Australia.

^3^Faculty of Health and Medical Sciences, The University of Adelaide, Adelaide, SA, Australia.

^4^Institute for Photonics and Advanced Sensing, School of Biological Sciences, The University of Adelaide, Adelaide, SA, Australia.

^5^Haematology Department, Royal Adelaide Hospital, Adelaide, SA, Australia.

^6^Australasian Leukaemia Lymphoma Group, Richmond, VIC, Australia.

^7^Australian and New Zealand Children’s Haematology-Oncology Group, Clayton, VIC, Australia.

**Supplemental Methods**

**Compounds**

ATP-competitive inhibitors (dasatinib, ponatinib), and the allosteric inhibitor asciminib, were purchased from Selleckchem (Houston, TX) and dissolved in DMSO (Sigma-Aldrich, St. Louis, MO) to a concentration of 10 mM. MK-4208 was provided by Terns Pharmaceuticals (Foster City, CA), dissolved in DMSO to a concentration of 10 mM. All inhibitors were stored at -80 °C.

**Cell line maintenance**

Ba/F3 cells were maintained in RPMI media (Thermo-Fisher, Waltham, MA) supplemented with 10% Foetal Calf Serum (FCS) and 5% WEHI-3B, as a source of murine IL-3. HEK-293T cells were maintained in DMEM (Thermo-Fisher) supplemented with 10% FCS. All culture media contained 2 mM L-glutamine, 20 Units/mL penicillin, and 20 mg/mL streptomycin sulfate. Both cell lines were cultured at 37°C/5% CO_2_ and were sourced from ATCC (Manassas, VA).

**RNA extraction for RT-qPCR**

Approximately 5x10^6^ cells were collected, washed with 1x PBS, pelleted, and lysed in 1 mL TRIzol reagent (Life Technologies, Carlsbad, CA). The samples were incubated at RT for 5 min, followed by the addition of 0.2 mL of chloroform (Sigma-Aldrich) and centrifugation at 12000 g for 15 min. RNA was precipitated by adding 20 μg of glycogen (Roche, Basel, CH) and 0.5 mL isopropanol (ChemSupply, Bedford St, SA), followed by incubation at RT for 10 min and centrifugation at 12000 g for 10 min. RNA pellets were washed with 75% ethanol and rehydrated in nuclease-free water (NF-H_2_0). RNA quality and quantity were measured on a Qubit 2.0 fluorometer (Invitrogen, Waltham, MA).

**cDNA synthesis**

Complementary DNA (cDNA) was synthesized utilizing the Quantitect Reverse Transcription Kit (Qiagen, Hilden, GER) using between 0.5-1 μg of RNA per sample.

**Polymerase Chain Reaction (PCR) & Site-Directed Mutagenesis (SDM)**

PCR was performed using 10-50 ng of DNA, 200 μM dNTPs (Invitrogen), 0.4-0.5 μM forward/reverse primers, 1-2 units Q5 High-Fidelity polymerase, 1x Q5 Reaction Buffer, 20% Q5 high GC Enhancer (New England Biolabs, Ipswich, MA) in a 25 μL reaction. The SDM primers were designed with the NEBaseChanger tool to generate the V299L, F311I, T315A/I and F317C/L mutations. The pRUFiG2-IRES-GFP-*BCR::ABL1* p190 and p210 vectors were used as a template. The Q5 Site-Directed Mutagenesis Kit (New England Biolabs) was used according to the manufacturer’s instructions. All reactions were performed in a T100 thermal cycler. Primers utilized for full-length PCR amplification and SDM are listed in Supplemental Table 1. PCR-amplified products were purified following the manufacturer’s protocol (QIAquick® Gel Extraction Kit, Qiagen). The purity and the concentration of the PCR samples were assessed using a Qubit 2.0 fluorometer (Invitrogen).

**Sanger Sequencing**

The purified PCR products, full-length *BCR::ABL1* p190 and p210, and mutants were sequenced using 30-1500 ng DNA (depending on the template type and length) and 400 nM of primer according to Australian Genome Research Facility (AGRF) recommendations. The resulting Sanger sequences were aligned to their respective reference sequences with Benchling (Biology Software, 2023, San Francisco, CA). Primers utilized for sequencing are listed in Supplemental Table 1.

**Plasmid preparation**

The *BCR::ABL1* sequences were purchased from Addgene (Watertown, MA) and subsequently cloned into the pRUFiG2 plasmid. The pRUFiG2 vectors containing the non-mutated *BCR::ABL1* p190, p210, and mutants (≥10 ng of DNA) were added to 50 μL of chemically competent DH5α *E. coli* (New England Biolabs) and incubated on ice for 30 min. The *E. coli* were then heat-shocked for 2 min at 42 °C and immediately placed on ice for 2 min, followed by the addition of 0.5-1 mL of Super Optimal broth with Catabolite repression (Thermo-Fisher), and incubation for 1 h at 37°C while shaking at 200 rpm. The *E.coli* were plated on agar plates with ampicillin (100 μg/mL) and incubated overnight at 37^o^C. Single colonies were selected and expanded in 50 mL of Luria Broth (LB) media containing ampicillin (100 μg/mL). Plasmids were extracted as previously described.^1^

**Transfection and transduction of Ba/F3 cells**

Retrovirus was produced by transient co-transfection of 6x10^5^ HEK-293T cells in 5 mL of appropriate media with 4 μg of pRUFiG2-*BCR::ABL1* fusion genes and 4 μg of pEQ-eco retroviral packaging vector. The transfections were performed in Opti-MEM media (Gibco, Waltham, MA) supplemented with 3-5% Lipofectamine 3000 (Invitrogen). The transfected HEK-293T cells were confirmed to express GFP by fluorescence microscopy before viral supernatant was collected and filtered through a 0.45 μm filter. The viral supernatant was utilized to transduce 1x10^6^ Ba/F3 cells by spinfection at 1800 rpm for 1 hour in the presence of 8 μg/mL Dextran-500 (TdB labs, Uppsala, Sweden). Polyclonal populations of cells harboring the *BCR::ABL1* fusion genes were sorted based on GFP positivity (>95% purity) with the BD FACSMelody^TM^ cell sorter (BD Biosciences, Franklin Lakes, NJ). The cell lines were generated once, and experiments represent biological replicates of each cell line.

**Real-time quantitative PCR (RT-qPCR)**

RT-qPCR was performed with SYBR green ROX qPCR reagents (Qiagen), following the manufacturer’s instructions. Data was quantified via Ct values, normalized to actin control, and presented as a 2^ΔΔCt^ fold change of expression. The qPCR primers (400 nM) are outlined in Supplemental Table 1.

**Western Blot**

Ba/F3 cells were washed with 1x PBS and lysed on ice for 10 min in 100 μL of NP40 buffer. Cell debris were pelleted via centrifugation, and the supernatant was collected. The 4X Laemmli’s Loading Buffer (Supplemental Table 3) was diluted to a final concentration of 1X and added to the supernatant. Cleared lysates were quantified using a DC protein assay according to the manufacturer’s instructions (Bio-Rad, Hercules, CA) and measured at 595 nm on a Bio-Rad iMark microplate reader (Bio-Rad). Protein lysates (40 μg) were separated using a 4-15% Criterion TGX stain-free precast gel (Bio-Rad), electrophoresed for 20 min at 100 V, followed by 1 h at 200 V and transferred to PVDF membrane using the High Molecular Weight settings on the TransBlot Turbo System (Bio-Rad). Membranes were blocked with Odyssey® Blocking Buffer (LI-COR Biosciences, Lincoln, NE) for 1 h at room temperature, followed by incubation with the primary antibody overnight. Membranes were washed in 1x TBST three times and stained with the secondary antibody for 1 h, washed 3x each in 1x TBST and then 1x TBS and subsequently imaged on the Odyssey CLx Imaging System (Li-COR). Reagents and antibodies used are shown in Supplemental Table 2 and 3.

**Annexin V/7-AAD cell death assays**

Transduced Ba/F3 cells were seeded at 4x10^4^ cells/mL in a 24-well plate and treated with vehicle control (DMSO) or increasing inhibitor concentrations ranging from 0 to 50000 nM for 72 h. Cells were washed with a binding buffer comprising of HANK’s Balanced Salt Solution (Sigma-Aldrich) supplemented with 5 mΜ calcium chloride (Sigma-Aldrich) and 1% HEPES (Sigma-Aldrich) and stained for 20 min in the dark with 0.4 µL AnnexinV-PE (BD Biosciences) and 0.04 μL 7-AAD (ThermoFisher) in 20 μL of binding buffer (Supplemental Table 2). The mean lethal dose required to kill 50% of cells (LD_50_) was calculated as a measure of treatment-induced cytotoxicity using GraphPad Prism software (GraphPad Software Inc., Version 9.5.1; La Jolla, CA).

**Flow cytometric analysis of downstream effector protein STAT5**

Ba/F3 cells were incubated for 2 h in 100 nM ponatinib, 5 μM asciminib, or 1 μM MK-4208 and fixed with a final concentration of 1.6% paraformaldehyde (ProSciTech, Kirwan, Qld). Drug concentrations were within the clinically achievable range of each inhibitor.^2,3^ The cells were washed in 1x PBS and permeabilized with ice-cold 80% methanol overnight at -20^o^C. Cells were resuspended in 1x PBS, 1x PBS/1% Bovine Serum Albumin (BSA), followed by staining in the dark at RT for 60 min with pSTAT5 or isotype control antibodies (Supplemental Table 2). The cells were washed in 1x PBS and analyzed on a BD FACSCanto™ Flow cytometry system (BD Biosciences).

**In silico modeling**

Point mutations V299L, F311I, T315A/I and F317C/L were modelled into the Abl structures bound to ATP-competitive TKIs dasatinib (PDB: 2GQG) and ponatinib (PDB: 3IK3), using ICM-Pro (MolSoft, San Diego, CA). **Note:** in the PDB: 3IK3 structure, the T315I mutation was corrected to wildtype using ICM-Pro software. Following the introduction of each point mutation, each model was subjected to local energy minimization and side chain refinement.^4-6^ The STAMP inhibitor, asciminib, was manually docked to the myristate pocket by transferring from the asciminib-bound structure (PDB: 5MO4), utilising ICM-Pro and energy-minimized with side-chain refinement. Structural analysis and visualization were performed using PyMOL (Schrödinger, Inc.).

**Quantification and statistical analysis**

FlowJo software (Version 10.8.1, FlowJo LLC, Ashland, OR) and GraphPad Prism software (Version 10.6.0) were used for experimental and statistical analysis. Unpaired Student’s T-test with Welch’s correction was utilized to determine the statistical difference between experimental groups. Differences were considered statistically significant when the p-value was <0.05 (*p<0.05, **p<0.01, ***p<0.001, ****p<0.0001).

| **Supplemental Table 1: Materials** | |
| --- | --- |
| **Primer Sequences** | |
| **pRUFiG2 vector** | |
| RCF-F | GGGGACTCTGCTGACCAC |
| IRES-R | ACACCGGCCTTATTCCAAG |
| **Ref. sequence *BCR* gene (NM_004327) and *ABL1* gene (NM_005157)** | |
| R1 | ATCGCTGCCGGTCATAGCTC |
| F1 | GAGGGTTCTCCGGGTAAGGC |
| R2 | TTCAACTCGGCGTCCTCGTAG |
| F2 | CTACCAGAGCATCTACGTCGGG |
| R3 | CATCGTTGGGCCAGATCTGC |
| F3 | CATTGATGACTCGCCCTCCTCA |
| R4 | CTTCTGGAAGAGGTCGCCCAC |
| F4 | GAGAACCTGAGAGCCAGAAGCA |
| R5 | AGTCATACTGCTGCGTTTTGCC |
| F5 | GCTGGACGCTTTGAAGATCAAGA |
| R6 | TGGCTGAGTGGACGATGACATT |
| F6 | GCTGGACCCAGTGAAAATGACC |
| R7 | GCTTGCCATCAGAAGCAGTGTT |
| F7 | AGCCCACTGTCTATGGTGTGTC |
| R8 | TGACGAGATCTGAGTGGCCATG |
| F8 | GGTAGCTGATTTTGGCCTGAGC |
| R9 | TGTTTCCCCAGCTCCTTTTCCA |
| F9 | CTGAGATGCCTCACTCCAAGGG |
| R10 | GCTTCCTGCGCTCTTGCTC |
| F10 | CACTGCCCTTTCCAGCTTCTTC |
| R11 | AGGGAGGGACAAGGGGAAATTG |
| F11 | CCGAAGAGACACTCGGGTTGAT |
| R12 | CTAGGAACTCCGAGCAGATGGC |
| F12 | TGTCACTGATTTCCTTCACCGA |
| **Site-Directed Mutagenesis (SDM): *BCR::ABL1* mutants** | |
| V299L_F | CCTAACCTG**ctg**CAGCTCCTTG |
| V299L_R | GTGTTTGATCTCTTTCATGACTGCAGC |
| F311I_F | GGAGCCCCCG**atc**TATATCATCA |
| F311I_R | CGGGTGCAGACCCCA |
| T315A_F | TCATC**gct**GAGTTCATGACCTAC |
| T315I_F | TCATC**att**GAGTTCATGACCTAC |
| T315_R | TATAGAACGGGGGCTCCC |
| F317C_F | CATCACTGAG**tgc**ATGACCTACG |
| F317L_F | CATCACTGAG**ctc**ATGACCTACG |
| F317_R | ATATAGAACGGGGGCTCCC |
| **RT-qPCR primers** | |
| *BCR::ABL1_*p190_F | CAGATCTGGCCCAACGAT |
| *BCR::ABL1_*p190_R | GAGCGGCTTCACTCAGACC |
| *BCR::ABL1_*p210_F | GGGGCTCTATGGGTTTCTG |
| *BCR::ABL1_*p210_R | ACCCTGAGGCTCAAAGTCAG |
| Actin F | GATCATTGCTCCTCCTGAGC |
| Actin R | GTCATAGTCCGCCTAGAAGCAT |

| **Supplemental Table 2: Antibodies List** | | |
| --- | --- | --- |
| **Protein** | **Conjugate** | **Manufacturer** |
| Annexin V | PE | BD, CAT #556421 |
| 7-AAD |  | Thermofisher, CAT #A1310 |
| IgG1 isotype | PE | BD, CAT #555909 |
| pSTAT5 | PE | BD, CAT #612567 |
| c-Abl Monoclonal Antibody |  | Thermofisher, CAT#MA5-14398 |
| β-Tubulin |  | CST #2128 (Danvers, MA) |
| donkey-anti-rabbit-IRDye | 680LT | Li-Cor, CAT # 926-68023 |
| donkey-anti-mouse-IRDye | 800CW | Li-Cor, CAT # 926-32212 |

| **Supplemental Table 3: Western Blotting Materials** | | |
| --- | --- | --- |
| **Reagent** | **Concentration** | **Company** |
| **4x Laemmli’s Loading Buffer** | | |
| Tris-HCL, pH 6.8 | 0.25 M | Sigma-Aldrich, CAT #252859 |
| SDS | 8% (w/v) | Sigma-Aldrich, CAT #75746 |
| Glycerol | 40% (v/v) | Sigma-Aldrich, CAT #G5516 |
| 2-mercaptoethanol | 20% (v/v) | Sigma-Aldrich, CAT #M6250 |
| Bromophenol blue | 0.05% (w/v) | Sigma-Aldrich, CAT #114405 |
| **NP40 Lysis Buffer Reagents** | | |
| Tris-HCL, pH 7.4 | 10 mM | Sigma-Aldrich, CAT #252859 |
| Glycerol | 10% | Sigma-Aldrich, CAT #G5516 |
| NP-40 (Igepal^TM)^ | 1% | Sigma-Aldrich, CAT #9002-93-1 |
| β-glycerol phosphate | 10 mM | Sigma-Aldrich, CAT #I8896 |
| Sodium Vanadate | 2 mM | Sigma-Aldrich, CAT #S6508 |
| Sodium Fluoride | 2 mM | Sigma-Aldrich, CAT #S7920 |
| Phenylmethylsulfonyl fluoride (PMSF) | 2 mM | Sigma-Aldrich, CAT #78830 |
| Sodium Pyrophosphate | 10 mM | Sigma-Aldrich, CAT #P8010 |
| Leupeptin | 1 μg/mL | Sigma-Aldrich, CAT #L0649 |
| Aprotinin | 5 μg/mL | Sigma-Aldrich, CAT #A1153 |
| Complete mini EDTA-free protease inhibitors Cocktail tablet | 1 per 10 mL | Roche, CAT #04693132001 |

**Supplemental Table 4: Inhibitor LD_90_ concentrations of Ba/F3 cells expressing BCR::ABL1 mutated p190 and p210 isoforms.**

|  | **Ponatinib (nM)** | | **Asciminib (nM)** | | **MK-4208 (nM)** | |
| --- | --- | --- | --- | --- | --- | --- |
| **Ba/F3 cell lines** | **LD_50_** | **LD_90_** | **LD_50_** | **LD_90_** | **LD_50_** | **LD_90_** |
| Empty vector | 586 | 816 | 23380 | 32359 | >50000 | >50000 |
| BCR::ABL1 **p190** | 0.75 | 1.1 | 4.9 | 16.7 | 6.1 | 70 |
| BCR::ABL1 **p210** | 0.53 | 0.74 | 5.2 | 22.3 | 5.1 | 19.2 |
| BCR::ABL1 **p190 V299L** | 1.49 | 2.2 | 16233 | 44014 | 692 | 46067 |
| BCR::ABL1 **p210 V299L** | 0.73 | 1.5 | 68.5 | 381 | 59.5 | 1032 |
| BCR::ABL1 **p190 F311I** | 2.45 | 3.8 | 6573 | 31541 | 488 | 39205 |
| BCR::ABL1 **p210 F311I** | 1.57 | 2.7 | 27.6 | 1437 | 22.9 | 99 |
| BCR::ABL1 **p190 T315A** | 1.35 | 1.9 | 56.6 | 5079 | 23.8 | 1525 |
| BCR::ABL1 **p210 T315A** | 1.03 | 1.6 | 15.8 | 148 | 13.1 | 1799 |
| BCR::ABL1 **p190 T315I** | 2.6 | 3.7 | 27195 | 36237 | 324 | 38440 |
| BCR::ABL1 **p210 T315I** | 2.35 | 3 | 35.4 | 98.5 | 16.8 | 40 |
| BCR::ABL1 **p190 F317C** | 1.54 | 3.1 | 22367 | 42580 | 8733 | 38417 |
| BCR::ABL1 **p210 F317C** | 1.32 | 2.1 | 132 | 1175 | 78.3 | 1197 |
| BCR::ABL1 **p190 F317L** | 2.65 | 3.8 | 14100 | 44105 | 615 | 36072 |
| BCR::ABL1 **p210 F317L** | 2.13 | 3.4 | 92.3 | 1208 | 64 | 853 |

**Supplemental Table 5: Inhibitor LD50 concentrations of Ba/F3 cells expressing BCR::ABL1 mutated p190 and p210 isoforms normalized to BCR::ABL1 protein expression.**

|  | **Ponatinib (nM)** | | **Asciminib (nM)** | | **MK-4208 (nM)** | |
| --- | --- | --- | --- | --- | --- | --- |
| **Ba/F3 cell lines (LD50s)** | Before | **After** | Before | **After** | Before | **After** |
| BCR::ABL1 **p190** | 0.75 | **0.39** | 4.9 | **2.53** | 6.1 | **3.1** |
| BCR::ABL1 **p210** | 0.53 | **0.44** | 5.2 | **4.3** | 5.1 | **4.22** |
| BCR::ABL1 **p190 V299L** | 1.49 | **1.18** | 16233 | **12852** | 692 | **547** |
| BCR::ABL1 **p210 V299L** | 0.73 | **0.64** | 68.5 | **60.3** | 59.5 | **52.4** |
| BCR::ABL1 **p190 F311I** | 2.45 | **1.9** | 6573 | **5111** | 488 | **380** |
| BCR::ABL1 **p210 F311I** | 1.57 | **1.4** | 27.6 | **24.9** | 22.9 | **20.7** |
| BCR::ABL1 **p190 T315A** | 1.35 | **0.88** | 56.6 | **36.9** | 23.8 | **15.5** |
| BCR::ABL1 **p210 T315A** | 1.03 | **0.73** | 15.8 | **11.2** | 13.1 | **9.3** |
| BCR::ABL1 **p190 T315I** | 2.6 | **2.44** | 27195 | **25583** | 324 | **304** |
| BCR::ABL1 **p210 T315I** | 2.35 | **2.24** | 35.4 | **33.7** | 16.8 | **16** |
| BCR::ABL1 **p190 F317C** | 1.54 | **1.09** | 22367 | **15826** | 8733 | **6179** |
| BCR::ABL1 **p210 F317C** | 1.32 | **1.13** | 132 | **113** | 78.3 | **67.3** |
| BCR::ABL1 **p190 F317L** | 2.65 | **2.16** | 14100 | **11529** | 615 | **502** |
| BCR::ABL1 **p210 F317L** | 2.13 | **1.97** | 92.3 | **85.7** | 64 | **59.5** |

**Before:** LD50 concentrations before normalization to BCR::ABL1 protein expression.

**After:** LD50 concentrations after normalization to BCR::ABL1 protein expression.

**Supplemental Figure 1: Computational modeling of BCR::ABL1 wildtype and ATP-pocket mutations.** X-ray crystal structures of Abl ATP-pocket bound with **A)** dasatinib (PDB: 2GQG; orange) or ponatinib (PDB: 3IK3; blue). ATP-pocket mutations **B)** V299L (pink), **C)** F311I (salmon), **D, E)** T315A/I (yellow) and **F, G)** F317C/L (purple) are highlighted. Substitutions at dasatinib contact residues **B)** V299L, **F)** F317C, and **G)** F317L abrogate inhibitor interactions, resulting in modest resistance to dasatinib. Residue T315 is a critical contact residue for dasatinib; substitution D) T315A results in a loss of a hydrogen bond with dasatinib, destabilizing the inhibitor, while substitution **E)** T315I sterically hinders dasatinib binding within the ATP-pocket.^7,8^ Residue T315A substitutions confer high levels of dasatinib resistance. Ponatinib binding remains largely unaffected by these mutations. **Note:** in the PDB 3IK3 structure, the T315I mutation was corrected to wild-type using ICM-Pro software.

**Supplemental Figure 2: The investigated ATP-pocket point mutations in their respective *BCR::ABL1* p190 or p210 isoforms.** Sanger sequencing confirmation of the site-directed mutagenesis generated point mutations on both *BCR::ABL1* isoforms. All fusion genes were completely sequenced, and no additional point mutations were identified. The predicted reference sequence is located at the top of each figure (*ABL1* NM_005157). The mutated residues are highlighted.

**Supplemental Figure 3:** **Confirmation of BCR::ABL1 expression in Ba/F3 cells. A)** RT-qPCR of RNA extracted from Ba/F3 cells confirmed the overexpression of *BCR::ABL1* fusion genes. The relative expression values were calculated using the housekeeping gene actin and normalized to Empty vector Ba/F3 cells. **B)** A total of 40 μg of protein extracted from Ba/F3 cells were denatured and electrophoresed on a 4-15% SDS-PAGE gel. Membranes were probed with primary antibodies specific for total Abl and β-tubulin (loading control). Empty vector cell lines were used as a negative control. BCR::ABL1 band intensity was normalized to β-tubulin. All experiments were performed in technical duplicates and (n=3) biological replicates. Columns represent the mean ±standard deviation, with individual data points shown. All experiments were performed with a minimum of three biological replicates and statistical significance was calculated using an unpaired Student's T-test with Welch’s correction (**p<0.05, **p<0.01*).

**Supplemental Figure 4: Efficacy of ATP-competitive TKI, dasatinib, against *BCR::ABL1* p190 and p210 isoforms and mutants.** Sensitivity of dasatinib was assessed by Annexin-V and & 7-AAD cell death assays. The mean LD_50_ nanomolar concentration of each cell line is denoted above its respective columns. Columns represent mean ±SD with individual data points shown. Red dotted lines represent the steady-state maximal plasma concentration of dasatinib (100 mg once daily): C_max_ =108 nM & C_trough_ =2.1 nM.^9^ All experiments were performed with a minimum of n =3 biological replicates. Statistical significance was calculated by Unpaired Student’s t-test with Welch’s correction, and significance is denoted with asterisks *(*p<0.05, **p<0.01, ***p<0.001).*

**References**

1.	Pronobis MI, Deuitch N, Peifer M. The Miraprep: A Protocol that Uses a Miniprep Kit and Provides Maxiprep Yields. *PLoS One*. 2016;11(8):e0160509.

2.	Hughes TP, Mauro MJ, Cortes JE, et al. Asciminib in Chronic Myeloid Leukemia after ABL Kinase Inhibitor Failure. *New England Journal of Medicine*. 2019;381(24):2315-2326.

3.	Cortes JE, Kantarjian H, Shah NP, et al. Ponatinib in refractory Philadelphia chromosome-positive leukemias. *N Engl J Med*. 2012;367(22):2075-2088.

4.	Härtel N, Klag T, Hanfstein B, et al. Enhanced ABL-inhibitor-induced MAPK-activation in T315I-BCR-ABL-expressing cells: a potential mechanism of altered leukemogenicity. *J Cancer Res Clin Oncol*. 2012;138(2):203-212.

5.	Abagyan R, Totrov M. Biased probability Monte Carlo conformational searches and electrostatic calculations for peptides and proteins. *J Mol Biol*. 1994;235(3):983-1002.

6.	Cardozo T, Totrov M, Abagyan R. Homology modeling by the ICM method. *Proteins*. 1995;23(3):403-414.

7.	Tokarski JS, Newitt JA, Chang CYJ, et al. The Structure of Dasatinib (BMS-354825) Bound to Activated ABL Kinase Domain Elucidates Its Inhibitory Activity against Imatinib-Resistant ABL Mutants. *Cancer Research*. 2006;66(11):5790-5797.

8.	Azam M, Nardi V, Shakespeare WC, et al. Activity of dual SRC-ABL inhibitors highlights the role of BCR/ABL kinase dynamics in drug resistance. *Proceedings of the National Academy of Sciences*. 2006;103(24):9244-9249.

9.	Rousselot P, Boucher S, Etienne G, et al. Pharmacokinetics of Dasatinib as a First Line Therapy In Newly Diagnosed CML Patients (OPTIM dasatinib trial): Correlation with Safety and Response. *Blood*. 2010;116(21):3432.
